# High complement protein C1q levels in pulmonary fibrosis and non-small cell lung cancer associated with poor prognosis

**DOI:** 10.1186/s12885-021-08912-3

**Published:** 2022-01-25

**Authors:** Wenxin Kou, Bo Li, Yeifei Shi, Yifan Zhao, Qing Yu, Jianhui Zhuang, Yawei Xu, Wenhui Peng

**Affiliations:** Department of Cardiology, Shanghai Tenth People’s Hospital, Tongji University School of Medicine, 301 Yanchang Road, Shanghai, 200072 China

**Keywords:** Complement, C1q, Idiopathic pulmonary fibrosis, Non-small-cell lung cancer, Prognosis, DNA methylation

## Abstract

**Background:**

Idiopathic pulmonary fibrosis (IPF) is the most common type of interstitial pneumonia. Lung cancer, mainly non-small cell lung cancer (NSCLC), is a complication of idiopathic pulmonary fibrosis. IPF is also an independent risk factor of lung cancer. Some studies have shown that the complement system can promote the progression of interstitial pulmonary fibrosis. In addition, C1q has also demonstrated to exert a tumor-promoting effect in many tumors. However, the role of C1q in idiopathic pulmonary fibrosis and lung cancer still remain unclear.

**Methods:**

We selected common differentially expressed genes in IPF and non-small cell lung cancer using datasets from GEO, and investigated common hub gene. The hub genes were validated in IPF by establishing mouse model of IPF and using another four datasets from the GEO. Multiple databases were analyzed including those of Kaplan–Meier Plotter, Tumor Immune Estimation Resource (TIMER2.0) and the Human Protein Atlas (HPA) for NSCLC.

**Results:**

In this study, 37 common DEGs were identified in IPF and NSCLC including 32 up-regulated genes and 5 down-regulated genes, and C1q was identified as common hub gene. The methylation status of C1q decreased and the expression levels of C1q increased in both lung cancer and idiopathic pulmonary fibrosis. The prognosis of non-small cell lung cancer and IPF patients with high levels of C1q is poor.

**Conclusions:**

These results show that C1q participates in pulmonary fibrosis and non-small cell lung cancer, and may be a potential diagnostic / prognostic biomarker or a therapeutic target.

**Supplementary Information:**

The online version contains supplementary material available at 10.1186/s12885-021-08912-3.

## Background

Idiopathic pulmonary fibrosis (IPF) is the most common type of interstitial pneumonia [1]. It is a fatal, invariably progressive disease. Lung transplantation is the most effective treatment [2]. The incidence of IPF appears to be increasing, so early recognition and intervention with supportive pharmacologic agents are needed to prevent its progression. Lung cancer (LC) is one of the most common complications in IPF patients, and mortality among patients with IPF and LC is highest [3]. Likewise, IPF is an independent risk factor for LC [4]. Nintedanib, used as an anti-fibrotic drug for IPF, is also approved for non-small cell lung cancer (NSCLC) [5], which further suggests the close relationship between IPF and NSCLC. Thus, the pathogenic overlap of IPF and LC may help clinicians to better understand the molecular mechanisms involved in both diseases, and may contribute to provide therapeutic strategies.

C1q protein was first identified in 1961 and was described as a “11s thermolabile serum protein” that participated in immune hemolysis [6]. It is the first subcomponent of the C1 complex, which activates the classical pathway of the complement [7]. C1q is composed of three polypeptide chains (C1qa, C1qb and C1qc) and regulates immune and non-immunes responses. The complement system appears to accelerate the pathogenesis of IPF [8, 9], while studies about the relationship between IPF and C1q are limited. C1q can act as a promoting factor of cancer in the tumor microenvironment [10]. However, the role of C1q in IPF and LC has not been fully explored.

**Fig. 1:**
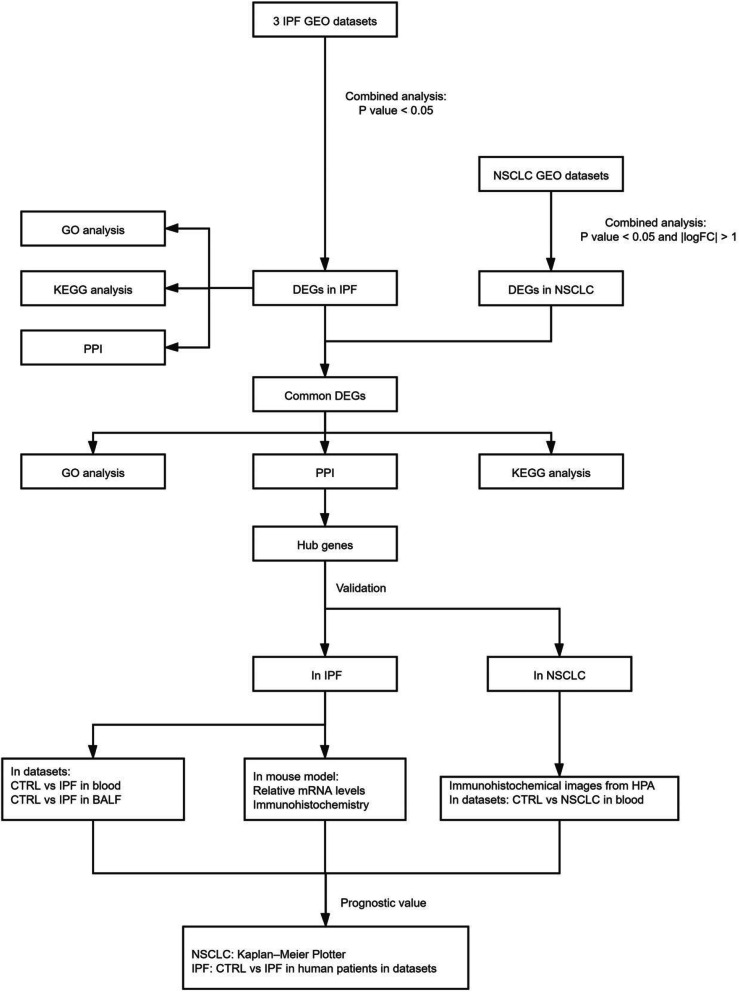
The flow diagram of this study

In our study, three mRNA microarray datasets were downloaded from the Gene Expression Omnibus (GEO) and 37 differentially expressed genes (DEGs) were obtained comparing lung tissue with pulmonary fibrosis and normal lung tissues. Next, we explored common DEGs in both IPF and NSCLC. Gene ontology (GO) terms and KEGG pathway enrichment analysis were performed using DAVID. Protein–protein interaction (PPI) network analysis was performed and hub genes were screened to aid better understand the relationships between DEGs. C1q was finally identified as a co-hub gene. We verified the role of C1q in IPF using another four datasets from the GEO and establish mouse model of IPF. Besides, we analyzed multiple databases including those of Kaplan–Meier Plotter, Tumor Immune Estimation Resource (TIMER2.0) and the Human Protein Atlas (HPA) to reveal the clinical significance and function of C1q in NSCLC especially in lung adenocarcinoma. Finally, SurvivalMeth platforms and GSE63704 was used to detecte methylation levels of C1q. The flow diagram of this study is shown in Fig. 1.

## Methods

### Mice

All experimental procedures involving animals were performed in accordance with the guidelines of the National Institutes of Health for the care and use of laboratory animals (NIH Publication, 8th Edition, 2011) and approved by the Animal Care and Use Committees of Shanghai Tenth People’s Hospital (Shanghai, China; permit number: SHDSYY-2019-2149). 12–16-week-old female mice were purchased from Charles River Laboratories. The murine pulmonary fibrosis model was induced with intratracheal injection of 1.5 U/kg BLM for 4 weeks, and control mice were given normal saline in same volume. After the study, all the mice were first anesthetized by allowing them to inhale 2.0% isoflurane and subjected to cervical dislocation. The study received approval by the Animal Care and Use Committees of Shanghai Tenth People’s Hospital for animal welfare.

### Real-time RT-PCR

Total RNA from lung tissue was extracted by Trizol reagent (Life Technologies), and then reserve transcribed to generate cDNA by the PrimeScript reverse transcription reagent kit (TaKaRa Bio, Shiga, Japan). Quantitative real-time PCR was carried out using SYBR Green (Roche). The gene Glyceraldehyde-3-phosphate dehydrogenase (GAPDH) was used as an internal control. Primers were listed in Supplemental Table 1.

### Histology and Immunohistology

Lung tissue samples were fixed by 4% of paraformaldehyde, dehydrated, and paraffin embedded for sectioning into 5-μm-thick sections and then used for Masson staining and immunohistochemical staining. The results of Masson staining were used to evaluate the cross-sectional area and the collagen volume respectively by light microscopy. Immunostainings for C1QA (A13659; ABclonal; 1:100), C1QB (A5339; ABclonal; 1:100) and C1QC (A9227; ABclonal; 1:100) was performed on paraffin embedded sections and incubated with goat anti-rabbit secondary antibodies to examine positive cells in lung tissue. All histopathological sections were measured with Image-Pro Plus 6.0 image analysis system.

### Microarray data

Data were retrieved from the GEO database, including the GSE485 series on the GPL81 platform (Affymetrix Murine Genome U74A Version 2 Array), the GSE97546 series on the GPL6887 platform (Illumina MouseWG-6 v2.0 expression beadchip), the GSE37635 [11] series on the GPL6885 platform (Illumina MouseRef-8 v2.0 expression beadchip), and the GSE31013 [12] series on the GPL1261 platform (Affymetrix Mouse Genome 430 2.0 Array). We chose samples from established animal models that required more than 1 week for fibrosis to be established to ensure the IPF model was successful (Table 1). Datasets including GSE98468, GSE102751 and GSE124685 [13, 14] were used to confirme the role of *C1q* in IPF. GSE63704 was used to detecte methylation levels of C1q in IPF. Datasets including GSE135304 and GSE76033 were used to confirme the role of *C1q* in NSCLC (Table 2).

**Table 1:** Characteristics of datasets for analysis

Dataset ID	Tissue	Strains of mice	Gender	Number of samples	GPL	References
GSE37635	Lung tissue	C57BL/6	Female	Control *n* = 7 1-week-IPF *n* = 7 2-week-IPF n = 6 3-week-IPF *n* = 6 4-week-IPF *n* = 6 5-week-IPF *n* = 6	GPL6885	Stoop R et al. 2013
GSE97546	Lung tissue	BALB/C	Female	Control *n* = 3 IPF *n* = 3	GPL6887	LeBleu V et al. 2020
GSE485	Lung tissue	C57BL/6	Male	Control *n* = 2 2-week-IPF *n* = 2	GPL81	Moller D et al. 2003
GSE31013	Lung tissue	B6C3F1 (C57BL/6 J × C3H F1)	Male	Control n = 6 Lung tumor *n* = 6	GPL1261	Sills RC et al. 2012

**Table 2:** Characteristics of the datasets for validation

Dataset ID	Tissue	Organism	NO. of samples	GPL	References
GSE98468	Balf	*Mus musculus*	Control *n* = 3 IPF *n* = 3	GPL19057	Xie N et al. 2017
GSE102751	Blood	*Homo sapiens*	Control *n* = 3 IPF *n* = 3	GPL11154	Habiel DM et al. 2019
GSE124685	Lung tissue	*Homo sapiens*	Control *n* = 35 IPF *n* = 49	GPL17303	Kaminiski N et al. 2019
GSE63704	Lung tissue	*Homo sapiens*	Control *n* = 37 IPF *n* = 43	GPL13534	Wielscher M et al. 2015
GSE135304	Blood	*Homo sapiens*	Control *n* = 88 NSCLC *n* = 404	GPL10558	Showe L et al. 2020
GSE76033	Blood	*Mus musculus*	Control *n *= 3 Lung cancer *n *= 3	GPL13112	Poczobutt JM et al. 2016

### Data processing of differentially expressed genes

GEO2R is an online tool that allows the comparison of groups in the GEO series to identify DEGs [15]. We identified DEGs comparing healthy mice and mice with lung fibrosis using GEO2R https://www.ncbi.nlm.nih.gov/geo/geo2r/ ). Genes without corresponding gene symbols were removed. Only one probe was kept for genes with different probes, and *p *< 0.05 was considered 143 for statistical significance. Co-differentially expressed *p *< 0.05 was considered for statistical significance. Co-differentially expressed genes were identified and Venn maps were drawn via the Venn online tool (http://bioinformatics.psb.ugent.be/webtools/Venn/ ).

### Functional enrichment analysis of differentially expressed genes 

DAVID ( https://david.abcc.ncifcrf.gov/ ) is an online bio-informatics tool composed of a comprehensive biological knowledge base and analytic tools. It can extract biological meaning of genes or proteins systematically [16]. We used DAVID to perform a functional enrichment analysis, including biological processes (BP), molecular functions (MF), and cellular components (CC), and KEGG pathway analysis of the overlapping DEGs( *p *< 0.05). The bubble chart was plotted by http://www.bioinformatics.com.cn , an online platform for data analysis and visualization.

### Construction of the protein interaction network and modules selection

The String online database (https://string-db.org) was used to construct a protein–protein interaction (PPI) network for DEGs and a combined-score > 0.4 was considered a significant difference. The PPI network was visualized with Cytoscape (www.cytoscape.org). Cytoscape has been used for integrated models of biomolecular interaction networks [17]. The essential proteins were predicted using the Molecular Complex Detection (MCODE) within the PPI network as follows: MCODE score > 5, degree cutoff = 2, node density cutoff = 0.1, node score cutoff = 0.2, k-score = 2, and maximum depth = 100.

### Survival analysis and validation of the hub genes

Kaplan–Meier Plotter (http://kmplot.com/analysis/index.php?p=service) was used to assess the prognostic value of hub genes in non-small-cell LC [18], which includes 1925 patients. We scanned the immunohistochemical images of lung adenocarcinoma and squamous cell carcinoma from The HPA (https://www.proteinatlas.org/), has open access allows free access to data for exploration of the human proteome [19]. TIMER2.0 [20] (http://timer.cistrome.org/) was used to analyze the correlation of C1q with the fibrosis indexes. SurvivalMeth (http://bio-bigdata.hrbmu.edu.cn/survivalmeth/) was used to analyze the effect of DNA methylation of C1q on LUAD prognosis [21].

### Statistical analysis

Microarray data was analysed using Statistical Package for Social Sciences (SPSS, Version 20.0). Heatmap, box plot and violin plot were drawn by Graphpad prism 8. Continuous variables were presented as mean ± SEM. Comparisons between groups were made using unpaired two-tailed t test or one-way ANOVA when appropriate and *p* < 0.05 was considered to be significant.

## Results

### Identification and analysis of differentially expressed genes in idiopathic pulmonary fibrosis

Three microarray datasets were chosen to identify DEGs in pulmonary fibrosis from the GEO. The GSE485 dataset contained 2 control samples and 2 pulmonary fibrosis samples. The GSE97546 dataset contained 3 lung samples of healthy mice and 3 lung samples of mice with lung fibrosis. The GSE37635 included 6 timepoints: the control (*n* = 7), 1 week (*n* = 7), 2 weeks (*n* = 6), 3 weeks (*n* = 6), 4 weeks (*n* = 6), and 5 weeks (*n* = 6) after bleomycin-treatment to induce lung fibrosis. We first extracted gene expressions changes at each time points in the GSE37635 dataset (Fig. S1), then the results were intersected with the other two datasets.

**Fig. 2:**
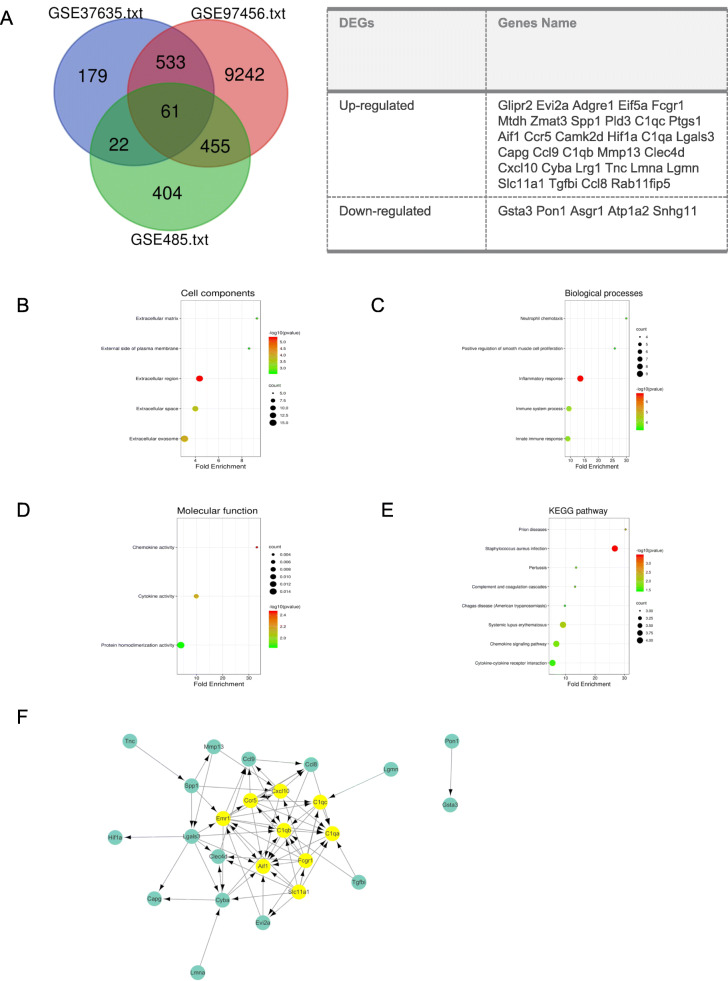
Analysis of DEGs in pulmonary fibrosis. (A) Venn diagram of DEGs in mRNA expression profiling datasets GSE485, GSE37635, and GSE97546. (B-D) GO and KEGG enrichment analysis of the DEGs. (F) PPI network of DEGs. (G) A significant module selected from PPI network, all of them were upregulated genes

Sixty one genes overlapped across the three datasets as shown in the Venn map. Genes with opposite trends were then excluded. Finally, we identified the 37 overlapping DEGs in the 3 datasets, consisting of 32 genes that were up-regulated and 5 genes that were down-regulated (Fig. 2A).

### Differentially expressed gene ontology and KEGG pathway analysis in pulmonary fibrosis

DAVID was used to perform GO term and KEGG pathway enrichment analysis for further insight into the function of the identified DEGs. We selected the top 5 terms of biological processes and cell components according to *p* value. We found cell components of DEGs mainly enriched in extracellular regions, extracellular exosome and extracellular space (Fig. 2B). The biological processes were mainly related to inflammation and immune pathways (Fig. 2C). Further, molecular function mainly involved chemokine activity, cytokine activity and protein homodimerization activity (Fig. 2D). Moreover, eight KEGG pathways were overrepresented in DEGs, including *staphylococcus aureus* infection, prion diseases, systemic lupus erythematosus, chemokine signaling pathway, pertussis, complement and coagulation cascades, cytokine-cytokine receptor interaction and chagas disease (Fig. 2E).

### Protein-protein interaction network construction and hub genes selection

The PPI network, which was consisted of 25 nodes and 74 edges, was constructed using STRING and was visualized by Cytoscape software to explore the association between the DEGs. The MCODE plugin was used to identify hub genes. *C1qa, Fcgr1, C1qb, C1qc, Ccr5, Slc11a1, Aif1, Emr1* and *Cxcl10* were identified as the most closely connected module which were highlighted in yellow, including 9 nodes and 32 edges (Fig. 2F), and the genes in this region were upregulated in pulmonary fibrosis.

### Screening and analysis of genes related to hub co-expressed genes in lung cancer

**Fig. 3:**
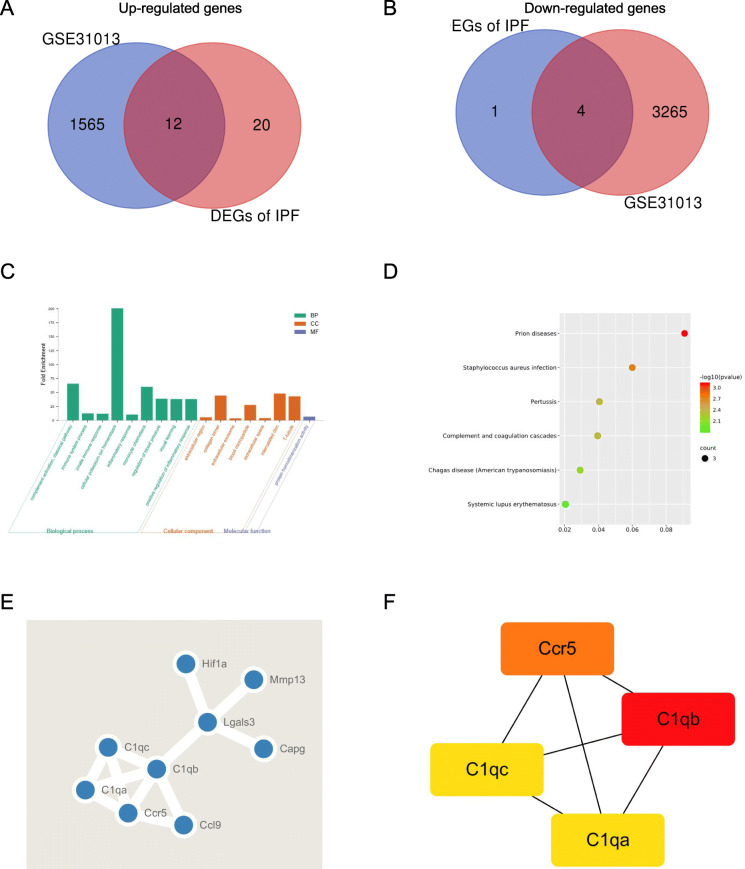
Analysis of commin DEGs in pulmonary fibrosis and non-small cell lung cancer. (A) Venn diagram of common DEGs in DEGs of IPF and GSE31013. (B) GO enrichment analysis of the common DEGs. (C) KEGG pathways of the common DEGs. (D) PPI network of common DEGs. (E) The most significant module related to common DEGs

Considering IPF increased the risk of LC development especially NSCLC [22], we selected the GSE31013 dataset containing 6 samples of lung tumors and 6 samples of age-matched normal lung tissue. After the DEGs between lung tumors and control samples were identified via GEO2R online tools with |logFC| > 1 and *p* value < 0.05, it was surprising to find that nearly half of the DEGs in pulmonary fibrosis also exhibited had significant difference in spontaneous lung tumors formations (Fig. 3A-B, Table S2).

The DEGs gene ontology and KEGG pathway of these genes were re-analyzed via DAVID to better understand their functions. The results showed that genes of the cellular component of this module were also mainly enriched in the extracellular region and the molecular function mainly enriched in protein homodimerization activity. The biological process analysis results suggested complement activation and cellular potassium ion homeostasis together with immune and inflammatory pathways might contribute to the occurrence and development of pulmonary fibrosis and LC (Fig. 3C-D). The top hub *(C1qa, C1qb, C1qc* and *Ccr5)* genes were also identified by the MCODE plugin, including 4 nodes and 6 edges (Fig. 3E-F).

### Validation of *C1q* in IPF and lung cancer

**Fig. 4:**
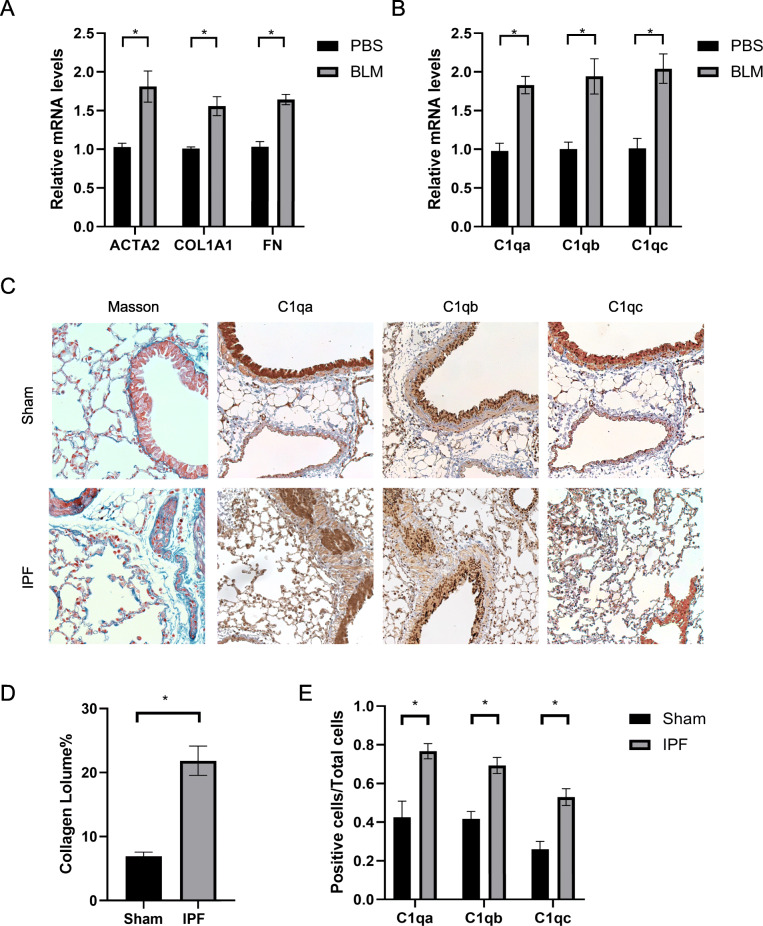
The expression of C1q (C1qa, C1qb, C1qc) in IPF. (A) Relative mRNA level analysis of fibrosis markers. (B) Relative mRNA level analysis of C1q. (C) Masson staining and immunostaining of C1q in IPF. *p < 0.05 versus Control group (*n* = 4 for each experimental group). All data are presented by mean ± SEM

As C1q ranked high among differentially expressed genes (Table S3), we focused on the role of *C1q* in IPF and LC. First, pulmonary fibrosis was induced by intratracheal instillation of bleomycin, while the control group was given normal saline in the same condition. The levels of fibrosis were measured by QPCR (Fig. 4A) and Masson staining (Fig. 4C-D) in IPF group increased, indicating the accomplishment of modeling. We further detected the levels of *C1q* by QPCR and immunohistochemistry, and found that the expression of *C1q* increased in IPF (Fig. 4B-C, E), consistent with previous analysis. Besides, we found the concentrations of C1q in the blood and balf of IPF increased as well (Fig. S2, Fig. S3).

**Fig. 5:**
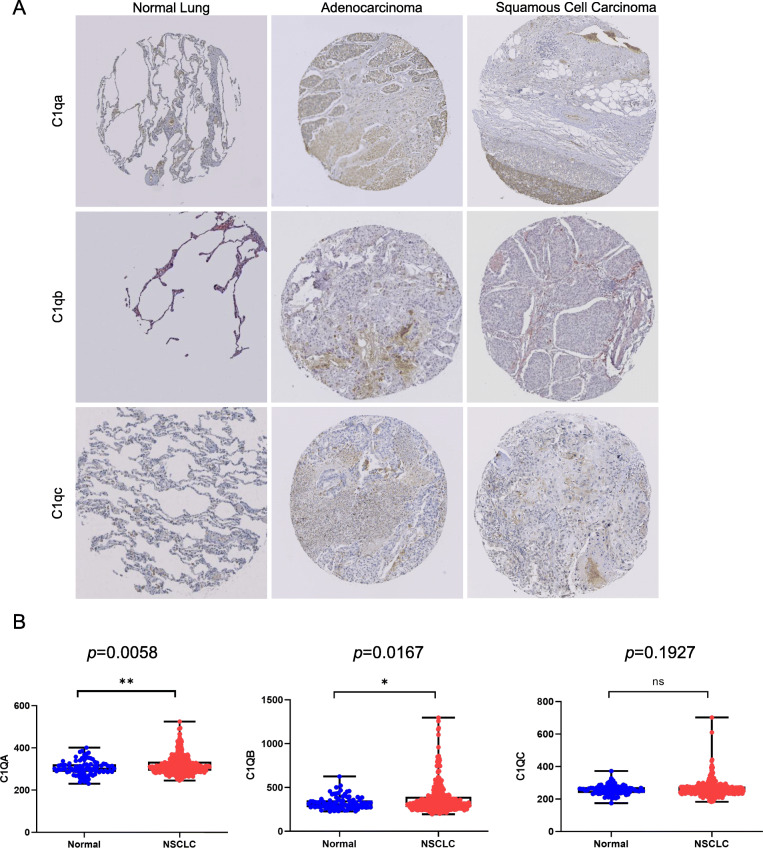
The expression of C1q (C1qa, C1qb, C1qc) in NSCLC including LUAD and LUSC. (A) Immunohistochemistry data of C1q (C1qa, C1qb and C1qc) in lung tissue of patients of control, LUAD and LUSC from The Human Protein Atlas. (B) The expression of C1q in the blood of patients with malignant (MN) pulmonary and patients with no nodules

The immunohistochemical stainings from the HPA showed *C1q* expression of lung adenocarcinoma was higher than that of normal lung, while squamous cell carcinoma showed no significance up-regulated trend (Fig. 5A, Fig. S4). Furthermore, data in GSE135304 revealed that the levels of C1q also increased in blood of patients with malignant (MN) pulmonary (Fig. 5B). To confirm the result in blood, we tested the expression of C1q in blood monocytes by GSE76033 as C1q mainly expresses in macrophages (Fig. S5, Fig. S6).

As methylation induced aberrant epigenetic regulation is an important common pathogenic mechanism of IPF and lung cancer [23], we explored DNA methylation levels of C1q in NSCLC (LUAD and LUSC) and IPF separately using the SurvivalMeth database and GSE63704. The results were shown in the box plot. We found most probes of hub genes decreased significantly in LUAD (*n* = 438) compared to normal samples (*n* = 32) (*p* < 0.05) (Fig. S7A-D). In LUSC, decreased probe of *C1q* also showed significant difference (Fig. S8A-D). Moreover, 4 probes of *C1qa*, 5 probes of *C1qb*, and 6 probes of *C1qc* were significantly decreased in IPF group (Fig. S9A-D). Common significant probes were listed in Table 3 and Table 4.

**Table 3:** Summary of significant methylation probes of hub genes

	LUAD		LUSC		IPF	
Gene	Up-regulated	Down-regulated	Up-regulated	Down-regulated	Up-regulated	Down-regulated
C1qa	0	5	0	6	0	9
C1qb	1	9	0	10	0	10
C1qc	1	8	2	8	0	9
Ccr5	1	4	1	4	0	4

**Table 4:** Characteristics of significant methylation probes of hub genes

Gene	Probe ID	UCSC RefGene Group	SNP
C1qa	cg11505417	3’UTR	rs1044378
	cg00108454	5’UTR	–
	cg17465569	5’UTR	–
	cg08710757	TSS1500	rs45557836
	cg10916651	TSS200	–
C1qb	cg18763854	3’UTR	–
	cg03941108	5’UTR	–
	cg22477971	5’UTR	–
	cg18366830	Body	rs11549682
	cg07012832	TSS1500	–
	cg10088685	TSS1500	–
	cg24931346	TSS200	–
	cg00215182	TSS200	–
C1qc	cg17104151	5’UTR	–
	cg00136477	5’UTR	–
	cg04161137	Body	–
	cg11152959	Body	rs45487196
	cg04161137	Body	–
	cg25600750	Body	–
	cg11393848	TSS1500	rs12404537
	cg12775742	TSS200	–
Ccr5	cg07616471	5’UTR	rs2856764
	cg00803692	TSS200	–
	cg04131610	TSS200	–

### The prognostic value of *C1q* in IPF and NSCLC

**Fig. 6:**
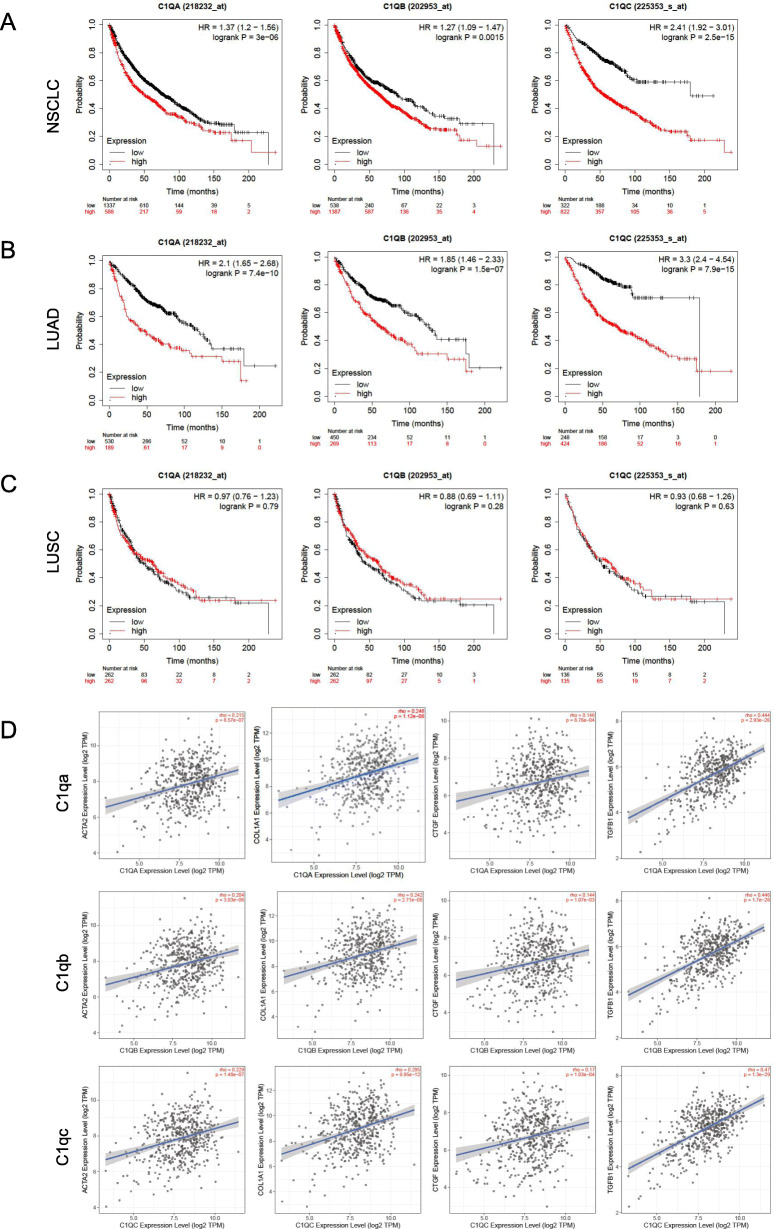
Overall survival analyses of C1q in NSCLC. (A) NSCLC patients with elevated C1q (C1qa, C1qb, C1qc) levels had lower OS (*p* < 0.05). (B) LUAD patients with elevated C1q levels had lower OS (*p* < 0.05). (C) The levels of C1q had no correlation with prognosis of LUSC. (D) C1q including C1qa, C1qb and C1qc were positive correlative with α-SMA, COL1A1, CTGF and TGF-β1 in lung adenocarcinoma at TIMER2.0 database

Expression of *C1qa, C1qb* and *C1qc* genes were examined and their clinical prognostic significance was investigated within the Kaplan–Meier Plotter database including 1925 cases of NSCLC. We noted that NSCLC patients with elevated *C1q (C1qa, C1qb, C1qc)* levels had lower OS (*p* < 0.05) (Fig. 6A). Patients with higher concentrations of *Ccr5* showed higher survival probability while it was positively correlated with collagen fiber related indexes, which was inconsistent with previous results, so we didn’t focus on the role of *Ccr5*(Fig. S10, Fig. S11). Furthermore, we investigated the Kaplan–Meier curve in 672 patients with lung adenocarcinoma (LUAD) and 271 patients with lung squamous cell carcinoma (LUSC). The results demonstrated that high levels of *C1q* expression revealed worse prognosis of adenocarcinoma (*p <* 0.05) (Fig. 6B) compared with squamous cell carcinoma (*p* > 0.05) (Fig. 6C).

We detected a relationship between *C1q* and *TGF-β1* as well as other mesenchymal markers (*ACTA2, COL1A1*, and *CTGF*) in lung adenocarcinoma using the online tool TIMER2.0. These factors involved in pulmonary fibrosis were positively related to the level of *C1q* expression (Fig. 6D).

**Fig. 7:**
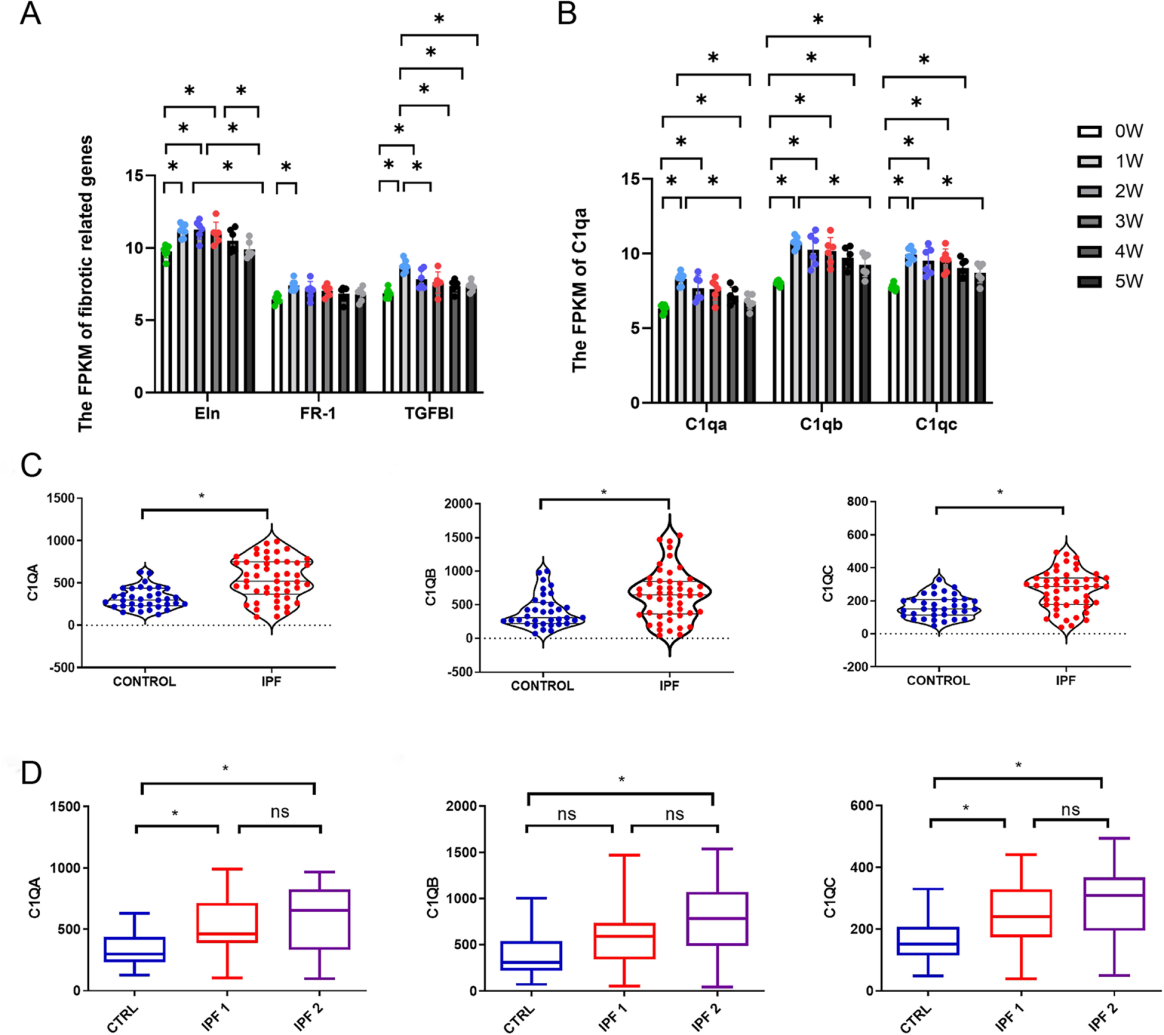
Overall survival analyses of C1q in IPF. (A) The variations of the expression of collagen fiber related markers including elastin (Eln), Fgfr1 (FR1) and TGFBI with time in IPF mice. (B) The variations of the expression of C1q with time in IPF mice. (C) The violin plot in expression of C1q between control and IPF. (D) The box plot in expression of C1q among control, IPF1 and IPF2

GSE37635 mentioned before was used to verify the role of C1q in IPF. It was found that elastin and collagen fiber related markers including *elastin* (Eln), *Fgfr1* (FR1) and *TGFBI* peaked at first week and decreased with time, in line with the expression of C1q (Fig. 7A, B). We further investigated it in IPF human patients by GSE124685 which had 84 samples including control samples (*n* = 35) and IPF samples (*n* = 49) from 10 control patients and 6 IPF patients (Table S4). The results were consistent with previous studies. The FPKM of C1q was also significantly increased in IPF patients (Fig. 7C). And then we divided samples IPF into two groups according to alveolar surface density (ASD) which was negatively correlated with degree of fibrosis. We identified samples with ASD > 6/μm as early or progressive fibrosis and grouped them to IPF1. Other samples (ASD < 6/μm) with end-stage fibrosis were grouped to IPF2. Although the difference between the IPF1 and IPF2 was not significant, the average expression levels of C1q in IPF2 group had an uptrend compared to IPF1 group (Fig. 7D, Table S5). These results suggested that C1q might also have prognostic value for IPF progression.

## Discussion

IPF is the most common of the idiopathic interstitial pulmonary disease. It is a fatal disease with a median survival of 4 years after diagnosis [24]. However, the underlying mechanisms and the treatment of IPF still remain unknown. Furthermore, growing epidemiologic evidence supports the association between IPF and LC. IPF can increase the risk of the development of LC by 7 to 20% [22]. Lung tumors in patients with IPF first occur adjacent to fibrotic areas. Compared with non-IPF-associated lung tumors, they have different histologic distribution and immunohistochemical features [23]. NSCLC is the predominant type in LC-IPF patients, of which adenocarcinoma (ADC) is the most common subtype [25]. Studies show that there are many mechanistic similarities between IPF and LC especially in NSCLC diseases, of which methylation is an important common mechanism [23, 26]. Myofibroblasts of IPF and cancer cells of LC are characterized by altered intercellular communication, enhanced migration and mobility, and increased invasion through the extracellular matrix. The fibroblasts in LC also exhibited mesenchymal-like features and have heterogeneous phenotypes [27].

In our study, a series of bioinformatics analyses were performed on three mRNA microarray datasets, and 37 common DEGs were identified including 32 up-regulated genes and 5 down-regulated genes. The cell component of DEGs was mainly enriched in the extracellular region and biological process were enriched in the inflammatory and immune responses, consistent with the previous study of IPF [28, 29]. It was found that among the DEGs, nine potential hub genes (*C1qa, Fcgr1, C1qb, C1qc, Ccr5, Slc11a1, Emr1, Aif1* and *Cxcl10*) were obtained using the MCODE plug-in of Cytoscape.

To explore the deep correlation between pulmonary fibrosis and NSCLC, we screened co-expressed genes in the DEGs between pulmonary fibrosis and LC, and surprisingly found 16 out of the 37 initial DEGs were also significantly more expressed in LC. Four hub genes for both IPF and NSCLC were finally identified (*C1qa, C1qb, C1qc, Ccr5*). *C1qa, C1qb,* and *C1qc* are three polypeptide chains the composed *C1q* which is the first subcomponent of the classical pathway of the complement system [30]. *Ccr5* is a chemokine receptor. It can mediate receptor activation by binding several endogenous chemokines [31].

We explored the methylation levels of hub genes, and found most of probes in IPF and NSCLC decreased which could give a good interpretation to the up regulated expression of *C1q*. In order to evaluate the diagnostic and prognostic value of the hub genes, we also determined the correlations between key genes and clinical data. The results demonstrated that the OS rate was significantly lower among NSCLC patients with high levels of *C1q*, while patients with high concentrations of *Ccr5* showed higher survival probability. We further investigated the role *C1q* played in the survival rate of lung adenocarcinoma and squamous cell carcinoma respectively. There was a significant difference in lung adenocarcinoma but no significant difference in lung squamous cell carcinoma, consistent with the immunohistochemical images analyzed from the PHA. Kaplan–Meier curves and immunohistochemical and stained pictures also indicated that *C1q* was closely related to the prognosis of NSCLC particularly lung adenocarcinoma. *TGF-β* is the most important mediator of the pathogenesis and carcinogenesis of IPF [32]. *C1q* showed a positive correlation with LUAD as well as other fibrosis markers like *a-SMA*, *CTGF* and *collagen-1*. These findings suggested that representative molecules contributing to IPF might also be regulated by *C1q* in lung adenocarcinoma. We further investigated the expression of *C1q* in IPF human patients, and found *C1q* might also have prognostic value for IPF progression. Besides, we comfirmed the role of *C1q* in the blood and balf of IPF.

*C1q* is synthesized primarily by macrophages and dendritic cells [33]. The complement component *C1q* plays an important role in host immune responses. Previous studies mainly focused on its role in systemic lupus erythematosus. Deficiency of *C1q* leads to high susceptibility to systemic lupus erythematosus-like symptoms, and complications with skin and renal diseases [34]. However, the role and mechanism of *C1q* in IPF and LC still remain unclear.

Correlation studies on the contribution to IPF are lacking. Sakkas et al. found that high levels of anti-*C1q* autoantibodies could predict the occurrence of pulmonary fibrosis patients with SSc [35]. Besides, *C1q* was positive in 73% of IPF cases by investigating bronchoalveolar lavage fluids, higher than in hypersensitivity pneumonitis (40%) or in sarcoidosis (31%) [36]. These result from another standpoint indicate that C1q participates in the occurrence and development of pulmonary fibrosis.

Studies accumulated over the past few years suggest that *C1q* can promote tumor formation by facilitating the proliferation and migration of cancer cells along with angiogenesis and metastasis [37, 38]. Bulla et al. reported that *C1q* promoted the growth of malignant pleural mesothelioma. *C1q* could induce adhesion and proliferation of mesothelioma cells and bind to hyaluronic acid in the microenvironment of MPM [39]. Tedesco et al. discovered that *C1q*-deficient (*C1qa*^*−/−*^) mice exhibited inhibited melanoma tumor growth which led to a better prognosis. C1q is mainly expressed in mesenchymal elements and promotes tumor growth cooperating with fibronectin [10]. Besides, *C1q* was also found to stimulate the progression of hepatocellular tumor. It enhanced migratory and invasive phenotypes of liver cancer cells [40]. Furthermore, *C1q* had a negative prognostic effect on kidney carcinomas [41].

*C1q* has pro-fibrotic and immunosuppressive properties, which are two contributing factors to both of IPF and NSCLC. *C1q* binding in human lung fibroblasts was reported to be heterogeneous [42], and able to interact with fibronectin to mediate adhesion of fibroblasts to immune complexes, resulting in stimulation in collagen synthesis [43, 44]. Low circulating levels of *C1q* prevented proliferation of fibroblast cells via down regulation of Wnt/β-catenin signaling [45, 46]. In addition, *C1q* plays a part in immune dysregulation. *C1q* inhibited proliferation of the Th1 and Th17 T cell subset mediated by human macrophage and dendritic cells [47]. It also suppressed the activation, proliferation, and cytotoxic functions of CD8^+^ T cells in vitro [48]. Meanwhile, tumor cells could directly assemble with *C1q*, enabling the formation of complement activation and further promoting tumor growth [49]. Other complement components like C4d and C5a showed similar phenomena in LC [50, 51]. Considering complement components have been demonstrated to promote tumor growth, the inhibition of complement activation and binding have been proposed as effective treatment for cancer [52, 53].

Our study revealed that the concentrations of *C1q* were increased and took part in many physiological and pathological processes in IPF and lung cancer. The results of bioinformatics analysis highlighted that high concentrations of *C1q* have important clinical prognostic value in NSCLC, especially in adenocarcinoma. The levels of *C1q* in severe pulmonary fibrosis also have an uptrend. It’s the first time that we find methylation status of *C1q* decreases and the expression of *C1q* increases in IPF and NSCLC, which is positively correlated with pulmonary fibrosis. This may also be a connection between the two diseases. In conclusion, our findings can provide the basis of new targets for diagnosis, therapy, and prognosis of IPF and LC.

## Supplementary Information

**Additional file 1: Supplementary Fig. 1.** Venn diagram of GSE37635. Common DEGs at 6 timepoints profiling GSE37635. **Supplementary Fig. 2.** The heatmap of GSE102751. It indicates the significant differences between the blood of control and IPF patients. The high expression and low expression are represented in red and green, respectively. **Supplementary Fig. 3.** The heatmap of GSE98468. It indicates the significant differences between the balf of control and IPF mice. The high expression and low expression are represented in red and green, respectively. **Supplementary Fig. 4.** Immunohistochemistry data of lung tissue. Immunohistochemistry data of C1q (C1qa, C1qb and C1qc) in normal lung, LUAD and LUSC from The Human Protein Atlas. **Supplementary Fig. 5.** The expression of C1q. C1q (C1qa, C1qb and C1qc) mainly express in macrophages in all cell types and lung tissue from The Human Protein Atlas. **Supplementary Fig. 6.** The expression of C1q in blood monocytes. The FPKM of C1q in blood monocytes significantly increased in 3-week tumor mice by GSE76033. **Supplementary Fig. 7.** DNA methylation levels of probes of hub genes in LUAD. (A) 5 methylation probes of C1qa showed significant difference in normal group and LUAD group; (B) 10 methylation probes of C1qb showed significant difference in normal group and LUAD group; (C) 9 methylation probes of C1qc showed significant difference in normal group and LUAD group;(D) 5 methylation probes of Ccr5 showed significant difference in normal group and LUAD group. **Supplementary Fig. 8.** Methylation levels of hub genes in LUSC. (A) 6 methylation probes of C1qa showed significant difference in normal group and LUSC group; (B) 10 methylation probes of C1qb showed significant difference in normal group and LUAD group; (C) 10 methylation probes of C1qc showed significant difference in normal group and LUSC group;(D) 4 methylation probes of Ccr5 showed significant difference in normal group and LUSC group. **Supplementary Fig. 9.** Methylation levels of hub genes in IPF. (A) 9 methylation probes of C1qa showed significant difference in normal group and IPF group; (B) 10 methylation probes of C1qb showed significant difference in normal group and IPF group; (C) 9 methylation probes of C1qc showed significant difference in normal group and IPF group;(D) 4 methylation probes of Ccr5 showed significant difference in normal group and IPF group. **Supplementary Fig. 10.** Overall survival analysis of Ccr5 in NSCLC patients. NSCLC patients with elevated Ccr5 levels had higher OS (p < 0.05). **Supplementary Fig. 11.** Relationship between Ccr5 and fibrosis in NSCLC. The expression of Ccr5 was positive correlative with α-SMA, COL1A, CTGF and TGF-β1 in NSCLC including LUAD and LUSC at TIMER2.0 database. **Supplemental Table 1.** Characteristics of the genes included in datasets. **Supplemental Table 2.** The logFC of DEGs. Up-regulated genes were labeled in red, down-regulated genes were labeled in black. **Supplemental Table 3** Characteristics of the genes included in datasets. **Supplemental Table 4** Characteristics of human subjects. **Supplemental Table 5** The means and standard deviations of FPKM of C1q.

## Data Availability

All the datasets are available in a public, open access repository. Databases Links GSE37635 https://www.ncbi.nlm.nih.gov/geo/query/acc.cgi?acc=GSE37635 GSE97546 https://www.ncbi.nlm.nih.gov/geo/query/acc.cgi?acc=GSE97546 GSE485 https://www.ncbi.nlm.nih.gov/geo/query/acc.cgi?acc=GSE485 GSE31013 https://www.ncbi.nlm.nih.gov/geo/query/acc.cgi?acc=GSE31013 GSE98468 https://www.ncbi.nlm.nih.gov/geo/query/acc.cgi?acc=GSE98468 GSE102751 https://www.ncbi.nlm.nih.gov/geo/query/acc.cgi?acc=GSE102751 GSE124685 https://www.ncbi.nlm.nih.gov/geo/query/acc.cgi?acc=GSE124685 GSE63704 https://www.ncbi.nlm.nih.gov/geo/query/acc.cgi?acc=GSE63704 Kaplan–Meier Plotter http://kmplot.com/analysis/index.php?p=service The HPA https://www.proteinatlas.org/ TIMER2.0 http://timer.cistrome.org/ SurvivalMeth http://bio-bigdata.hrbmu.edu.cn/survivalmeth/

## Abbreviations

IPF: Idiopathic pulmonary fibrosis
LC: Lung cancer
NSCLC: Non-small cell lung cancer
GEO: Gene expression omnibus
DEGs: Differentially expressed genes
GO: Gene ontology
KEGG: Kyoto encyclopedia of genes and genomes
DAVID: Database for annotation, visualization and integrated discovery
PPI: Protein–protein interaction
TIMER2.0: Tumor immune estimation resource2.0
HPA: The human protein atlas
BP: Biological processes
MF: Molecular functions
CC: Cellular components
MCODE: The molecular complex detection
QPCR: Quantitative real-time PCR
LUAD: Lung adenocarcinoma
LUSC: Lung squamous cell carcinoma
OS: Overall survival
FPKM: Fragments per Kilobasekof exon model per million mapped fragments
ASD: Alveolar surface density
ADC: Adenocarcinoma
MPM: Malignant pleural mesothelioma
