# Supplementary material for: High complement protein C1q levels in pulmonary fibrosis and non-small cell lung cancer associated with poor prognosis
Source: BMC Cancer. 2022 Jan 25;22:110. doi: 10.1186/s12885-021-08912-3 (PMC8790889; doi:10.1186/s12885-021-08912-3)
Supplement: Supplementary file 1 — Additional file 1: Supplementary Fig. 1. Venn diagram of GSE37635. Common DEGs at 6 timepoints profiling GSE37635. Supplementary Fig. 2. The heatmap of GSE102751. It indicates the significant differences between the blood of control and IPF patients. The high expression and low expression are represented in red and green, respectively. Supplementary Fig. 3. The heatmap of GSE98468. It indicates the significant differences between the balf of control and IPF mice. The high expression and low expression are represented in red and green, respectively. Supplementary Fig. 4. Immunohistochemistry data of lung tissue. Immunohistochemistry data of C1q (C1qa, C1qb and C1qc) in normal lung, LUAD and LUSC from The Human Protein Atlas. Supplementary Fig. 5. The expression of C1q. C1q (C1qa, C1qb and C1qc) mainly express in macrophages in all cell types and lung tissue from The Human Protein Atlas. Supplementary Fig. 6. The expression of C1q in blood monocytes. The FPKM of C1q in blood monocytes significantly increased in 3-week tumor mice by GSE76033. Supplementary Fig. 7. DNA methylation levels of probes of hub genes in LUAD. (A) 5 methylation probes of C1qa showed significant difference in normal group and LUAD group; (B) 10 methylation probes of C1qb showed significant difference in normal group and LUAD group; (C) 9 methylation probes of C1qc showed significant difference in normal group and LUAD group;(D) 5 methylation probes of Ccr5 showed significant difference in normal group and LUAD group. Supplementary Fig. 8. Methylation levels of hub genes in LUSC. (A) 6 methylation probes of C1qa showed significant difference in normal group and LUSC group; (B) 10 methylation probes of C1qb showed significant difference in normal group and LUAD group; (C) 10 methylation probes of C1qc showed significant difference in normal group and LUSC group;(D) 4 methylation probes of Ccr5 showed significant difference in normal group and LUSC group. Supplementary Fig. 9. Methylation levels of [file 12885_2021_8912_MOESM1_ESM.docx]

**High Complement Protein C1q levels in Pulmonary Fibrosis and Non-small Cell Lung Cancer Associated with Poor Prognosis**

Wenxin Kou^1, *^, Bo Li^1, *^, Yeifei Shi^1^, Yifan Zhao^1^, Qing Yu^1^, Jianhui Zhuang^1^, Yawei Xu^1, #^ , Wenhui Peng^1,#^

1 Department of Cardiology, Shanghai Tenth People’s Hospital, Tongji University School of Medicine, Shanghai, China

* Contributed equally to this work

# Corresponding author:

Yawei XU, MD, PhD and Wenhui Peng, MD, PhD, Shanghai Tenth People’s Hospital, Tongji University School of Medicine, 301 Yanchang Road, Shanghai 200072, China

E-mail: xuyawei@tongji.edu.cn, pwenhui@tongji.edu.cn

Fax: 086-21-66301051

Phone: 086-21-66307259

**
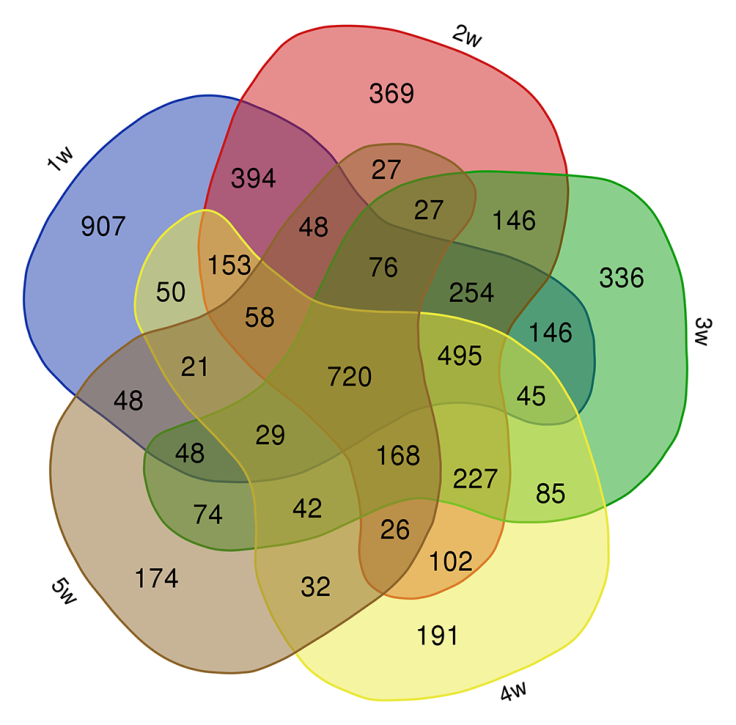
**

**Supplementary Figure 1** Venn diagram of GSE37635. Common DEGs at 6 timepoints profiling GSE37635.


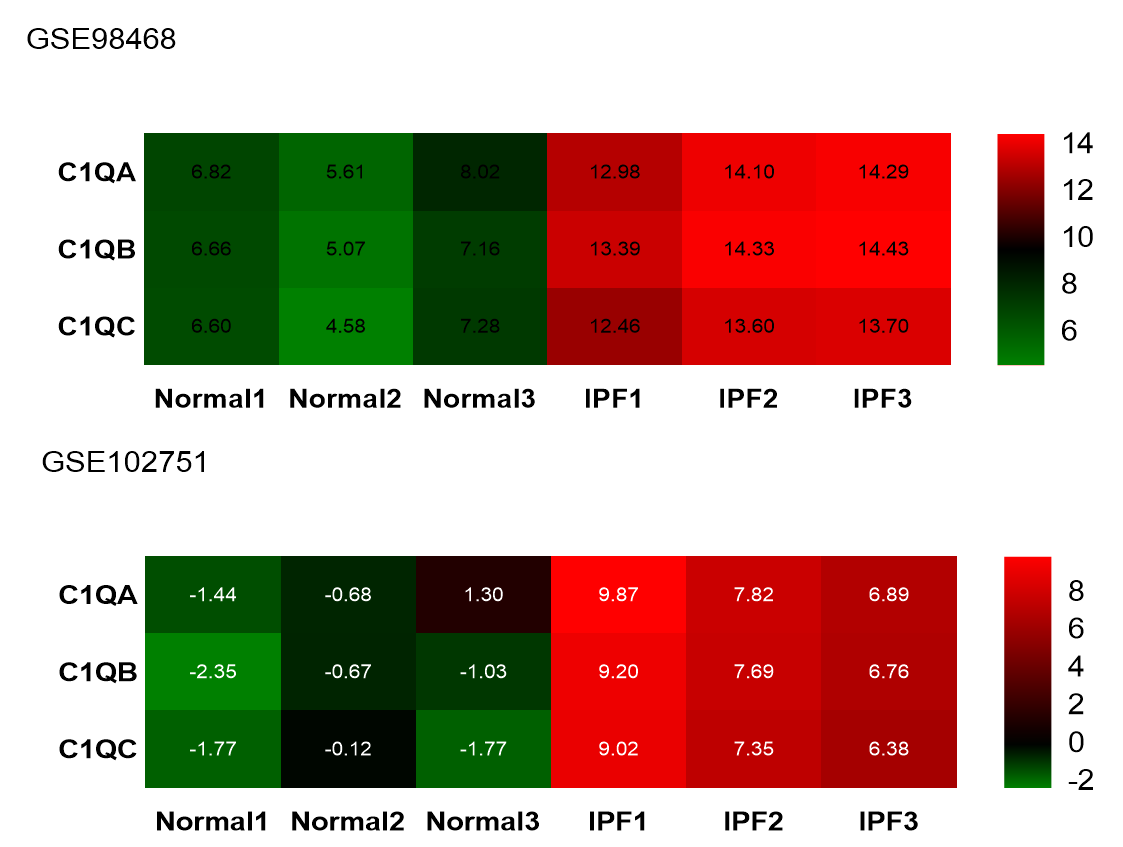


**Supplementary Figure 2** The heatmap of GSE102751. It indicates the significant differences between the blood of control and IPF patients. The high expression and low expression are represented in red and green, respectively.


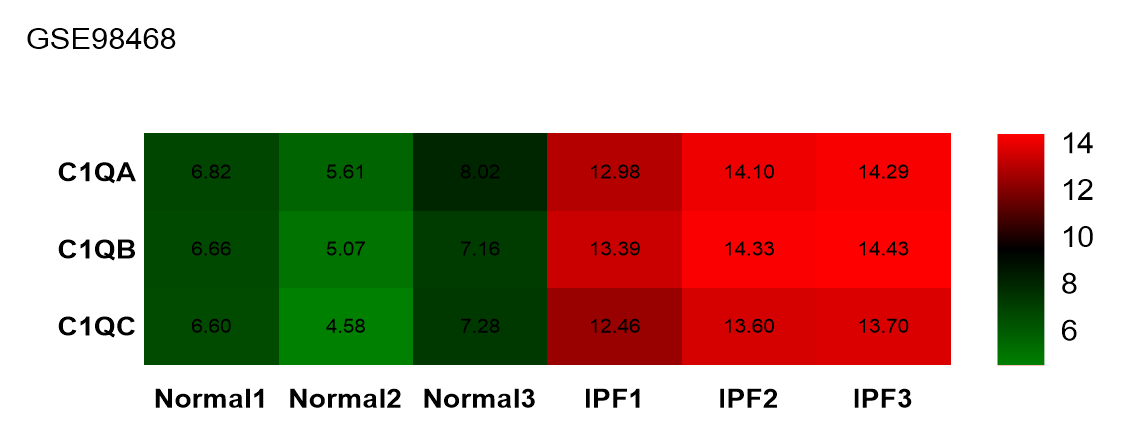


**Supplementary Figure 3** The heatmap of GSE98468. It indicates the significant differences between the balf of control and IPF mice. The high expression and low expression are represented in red and green, respectively.

**
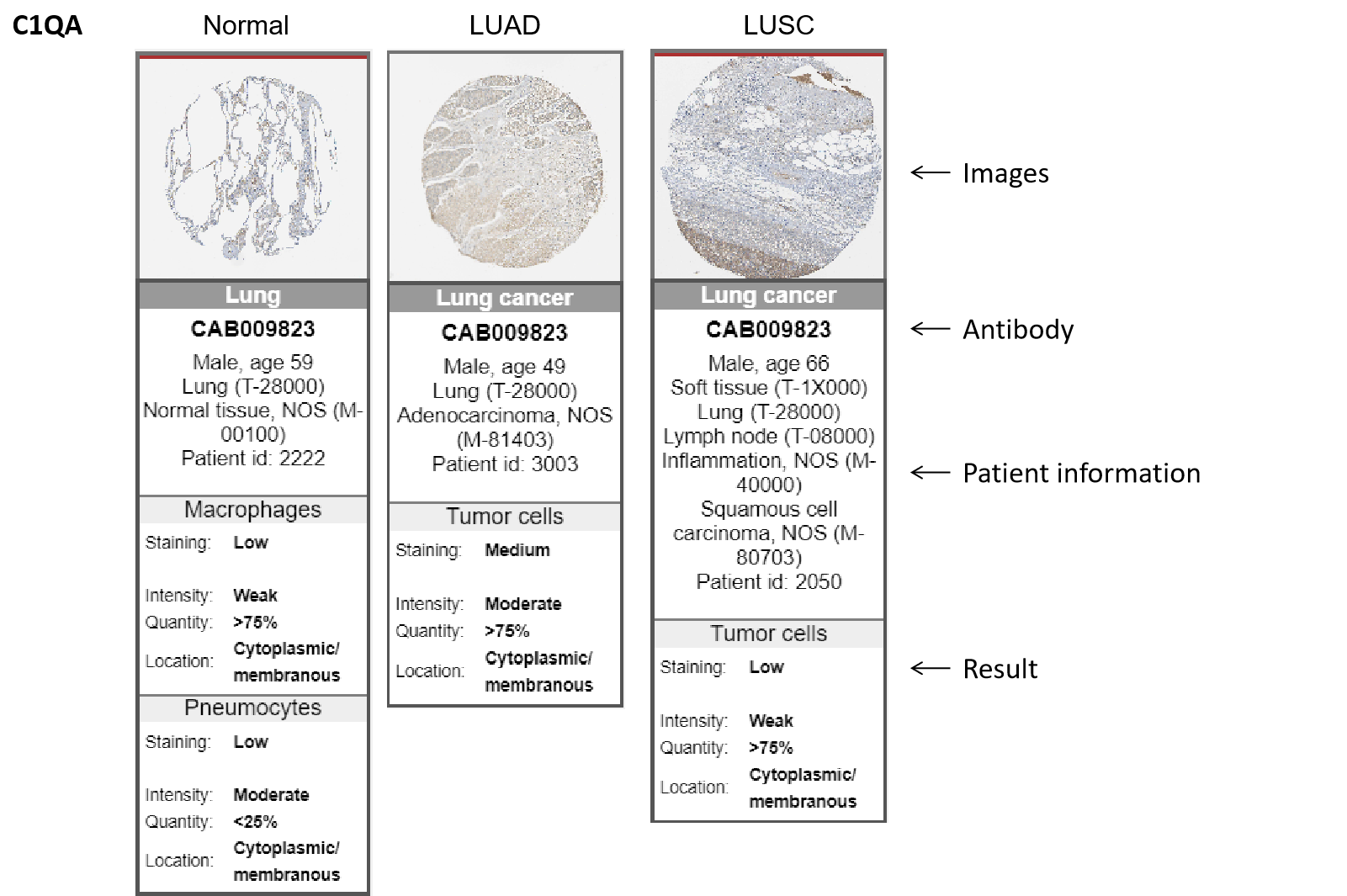
**

**
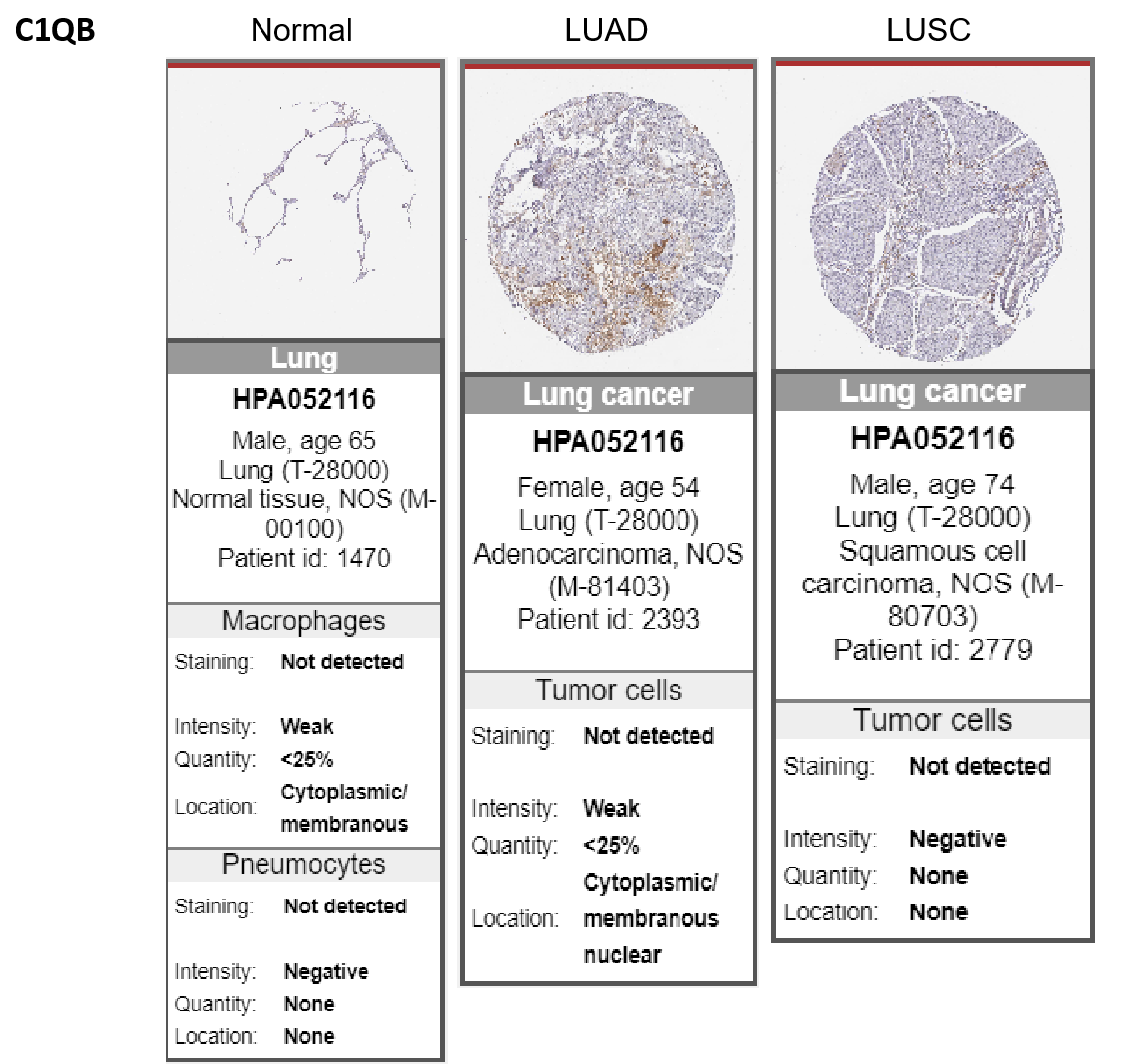
**

**
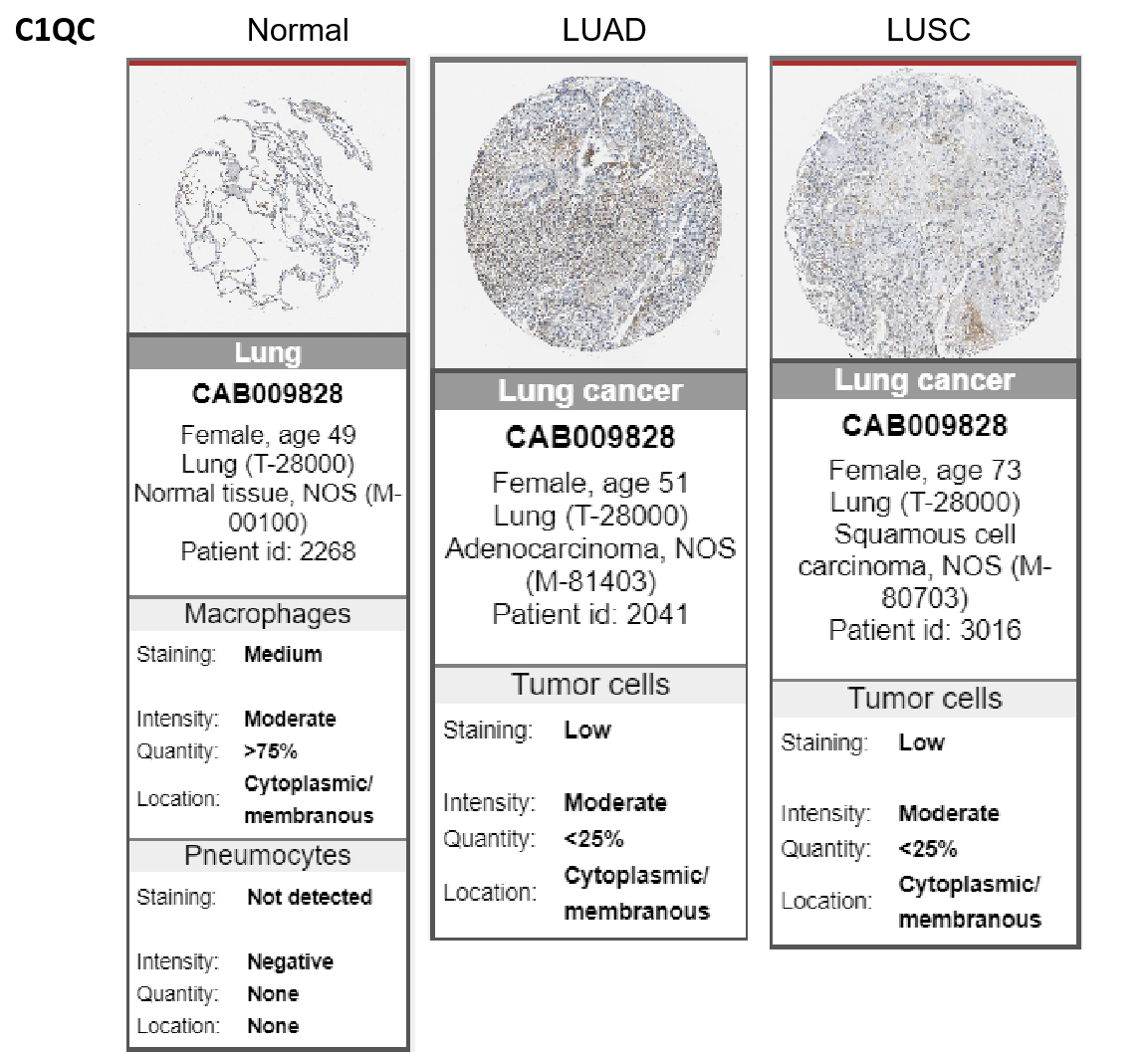
**

**Supplementary Figure 4** Immunohistochemistry data of lung tissue. Immunohistochemistry data of C1q (C1qa, C1qb and C1qc) in normal lung, LUAD and LUSC from The Human Protein Atlas.

**Supplementary Figure 5** The expression of *C1q*. *C1q* (*C1qa*, *C1qb* and *C1qc*) mainly express in macrophages in all cell types and lung tissue from The Human Protein Atlas.

.

**Supplementary Figure 6** The expression of C1q in blood monocytes. The FPKM of C1q in blood monocytes significantly increased in 3-week tumor mice by GSE76033.

**
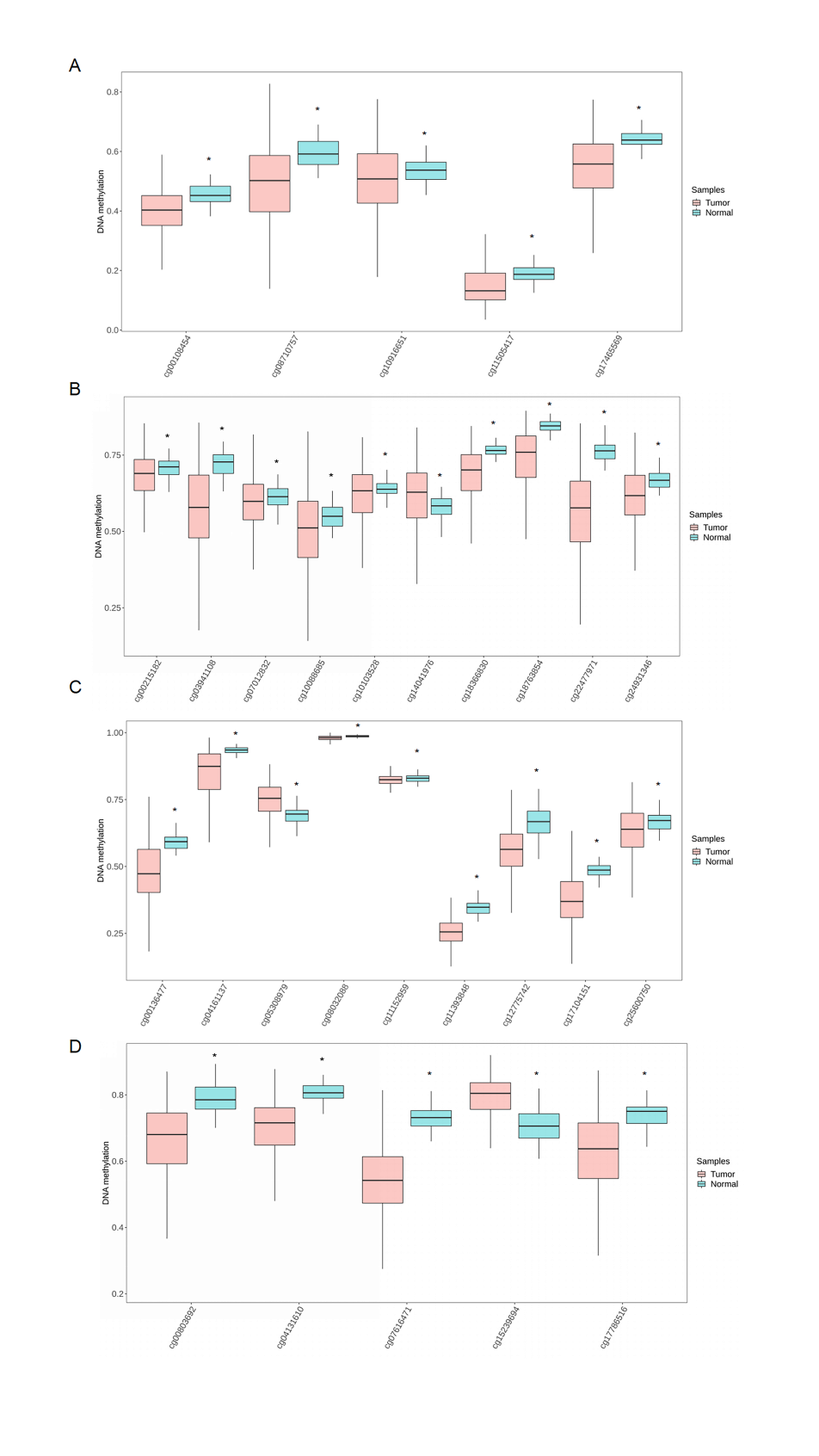
**

**Supplementary Figure 7** DNA methylation levels of probes of hub genes in LUAD. (A) 5 methylation probes of C1qa showed significant difference in normal group and LUAD group; (B) 10 methylation probes of C1qb showed significant difference in normal group and LUAD group; (C) 9 methylation probes of C1qc showed significant difference in normal group and LUAD group;(D) 5 methylation probes of Ccr5 showed significant difference in normal group and LUAD group.

**
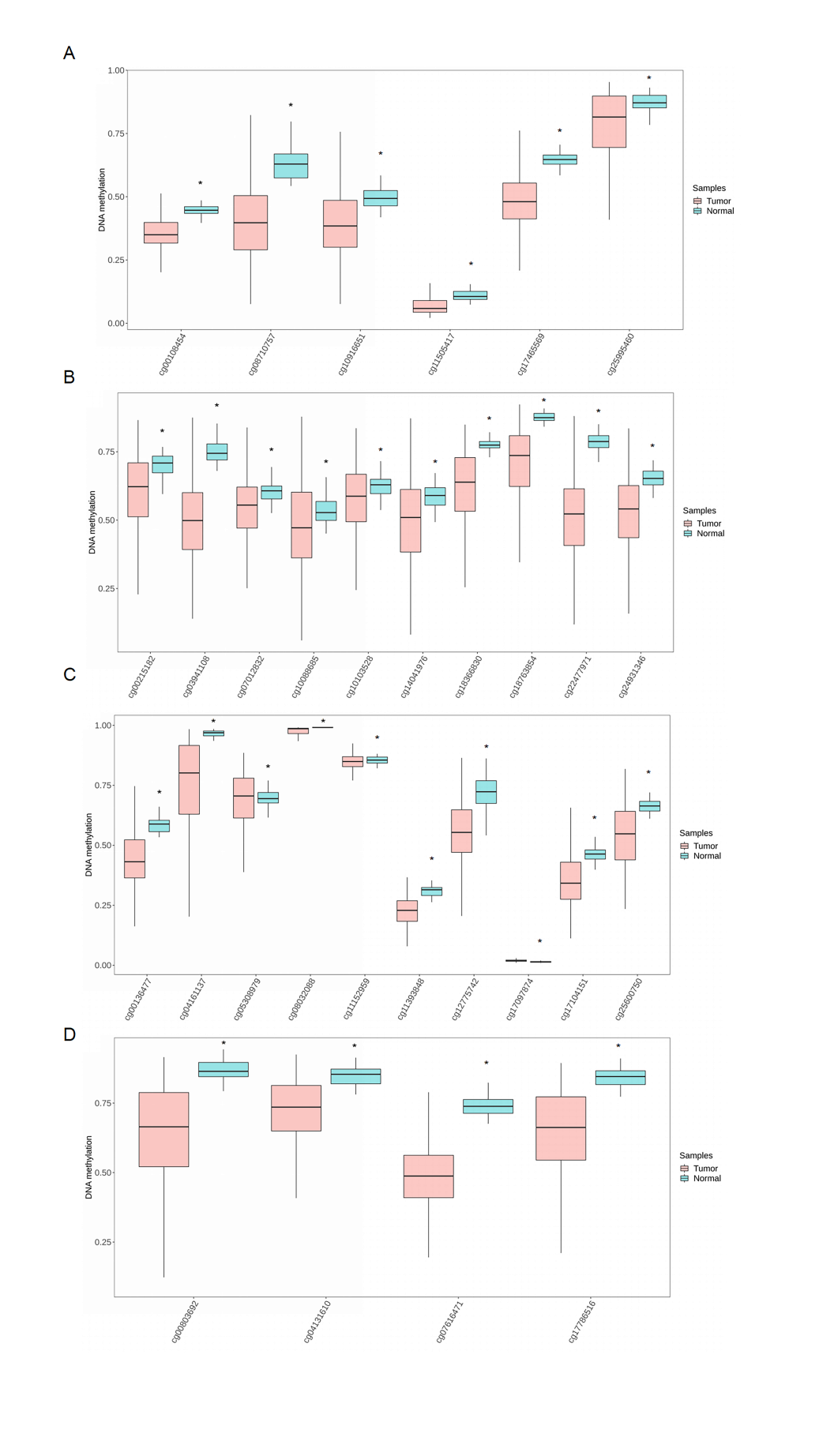
**

**Supplementary Figure 8** Methylation levels of hub genes in LUSC. (A) 6 methylation probes of C1qa showed significant difference in normal group and LUSC group; (B) 10 methylation probes of C1qb showed significant difference in normal group and LUAD group; (C) 10 methylation probes of C1qc showed significant difference in normal group and LUSC group;(D) 4 methylation probes of Ccr5 showed significant difference in normal group and LUSC group.

**
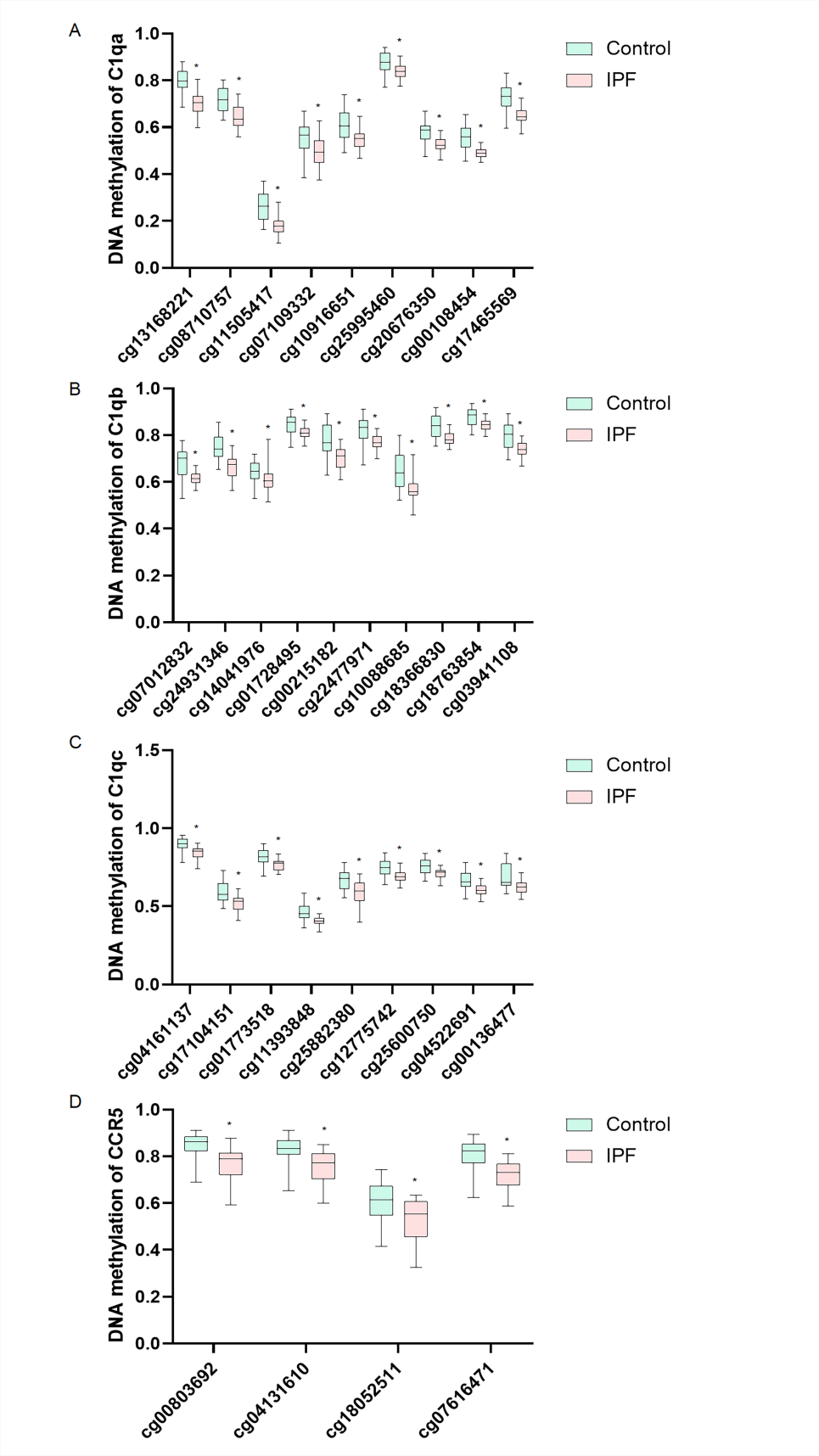
**

**Supplementary Figure 9** Methylation levels of hub genes in IPF. (A) 9 methylation probes of C1qa showed significant difference in normal group and IPF group; (B) 10 methylation probes of C1qb showed significant difference in normal group and IPF group; (C) 9 methylation probes of C1qc showed significant difference in normal group and IPF group;(D) 4 methylation probes of Ccr5 showed significant difference in normal group and IPF group.

**Supplementary Figure 10** Overall survival analysis of Ccr5 in NSCLC patients. NSCLC patients with elevated Ccr5 levels had higher OS (p < 0.05)

**Supplementary Figure 11** Relationship between Ccr5 and fibrosis in NSCLC. The expression of Ccr5 was positive correlative with α-SMA, COL1A, CTGF and TGF-β1 in NSCLC including LUAD and LUSC at TIMER2.0 database.

**Supplemental Table 1** Characteristics of the genes included in datasets

| **Gene Name** | **Primer** | **Sequence** |
| --- | --- | --- |
| **C1QA -MS** | Formate 5’-3’ | GGACTGGTATCCGAGGTTTTAA |
|  | Reverse 5’-3’ | GATATTGCCTGGATTGCCTTTC |
| **C1QB -MS** | Formate 5’-3’ | CTACACAGAAAGTCGCCTTCTC |
|  | Reverse 5’-3’ | CTGGCATGATAGGTGAAGTAGT |
| **C1QC -MS** | Formate 5’-3’ | TACTTCGTCTACTACACATCGC |
|  | Reverse 5’-3’ | GGAAACAGTAGGAAACCAGAGA |
| **ACTA2 -MS** | Formate 5’-3’ | GTCCCAGACATCAGGGAGTAA |
|  | Reverse 5’-3’ | TCGGATACTTCAGCGTCAGGA |
| **COL1A1 -MS** | Formate 5’-3’ | TAAGGGTCCCCAATGGTGAGA |
|  | Reverse 5’-3’ | GGGTCCCTCGACTCCTACAT |
| **FN1 -MS** | Formate 5’-3’ | ATGTGGACCCCTCCTGATAGT |
|  | Reverse 5’-3’ | GCCCAGTGATTTCAGCAAAGG |
| **GAPDH -MS** | Formate 5’-3’ | AGGTCGGTGTGAACGGATTTG |
|  | Reverse 5’-3’ | TGTAGACCATGTAGTTGAGGTCA |

**Supplemental Table 2** The logFC of DEGs. Up-regulated genes were labeled in red, down-regulated genes were labeled in black.

| Genes | 1W | 2W | 3W | 4W | 5W | GSE485 | GSE97546 | GSE31013 |
| --- | --- | --- | --- | --- | --- | --- | --- | --- |
| Pon1 | -0.71 | -1.07 | -0.90 | -0.72 | -0.38 | -2.16 | -2.53 | -4.91 |
| Asgr1 | -2.79 | -2.53 | -2.30 | -1.61 | -1.23 | -2.56 | -3.58 | -4.75 |
| Atp1a2 | -1.19 | -0.90 | -0.78 | -0.56 | -0.45 | -1.61 | -2.02 | -3.38 |
| Snhg11 | -1.59 | -1.42 | -1.04 | -0.80 | -0.32 | -1.47 | -1.87 | -1.79 |
| Pld3 | 1.25 | 1.27 | 1.19 | 0.75 | 0.68 | 1.01 | 2.32 | 1.10 |
| C1qc | 2.19 | 1.77 | 1.78 | 1.28 | 0.95 | 2.28 | 3.25 | 1.11 |
| Ptgs1 | 1.27 | 0.94 | 0.71 | 0.81 | 0.62 | 0.77 | 1.91 | 1.14 |
| Ccr5 | 3.86 | 3.23 | 2.50 | 2.57 | 1.81 | 2.78 | 3.51 | 1.17 |
| Camk2d | 1.08 | 1.12 | 1.13 | 1.07 | 1.15 | 1.00 | 3.03 | 1.29 |
| Hif1a | 1.38 | 2.15 | 1.65 | 1.09 | 1.15 | 0.72 | 3.25 | 1.35 |
| C1qa | 1.98 | 1.36 | 1.30 | 0.88 | 0.45 | 2.00 | 2.64 | 1.38 |
| Lgals3 | 1.13 | 0.72 | 0.52 | 0.51 | 0.49 | 1.11 | 2.27 | 1.39 |
| Capg | 1.09 | 0.68 | 0.76 | 0.54 | 0.48 | 3.13 | 1.74 | 1.40 |
| Ccl9 | 2.64 | 1.98 | 1.84 | 1.27 | 0.82 | 2.44 | 3.14 | 1.61 |
| C1qb | 2.73 | 2.24 | 2.14 | 1.69 | 1.22 | 3.07 | 2.88 | 2.04 |
| Mmp13 | 2.24 | 2.12 | 1.64 | 1.43 | 0.95 | 2.05 | 4.45 | 2.76 |
| Gsta3 | -0.89 | -0.88 | -0.70 | -0.51 | -0.81 | -0.91 | -1.59 |  |
| Adgre1 | 1.82 | 1.18 | 1.29 | 1.14 | 0.62 | 1.49 | 2.83 |  |
| Eif5a | 0.60 | 0.80 | 0.66 | 0.58 | 0.44 | 1.94 | 1.74 |  |
| Fcgr1 | 2.41 | 1.07 | 1.17 | 0.93 | 1.27 | 1.13 | 3.59 |  |
| Mtdh | 0.54 | 0.60 | 0.33 | 0.36 | 0.32 | 1.22 | 1.91 |  |
| Zmat3 | 0.82 | 0.84 | 0.96 | 0.81 | 0.77 | 0.77 | 1.88 |  |
| Spp1 | 3.04 | 2.46 | 2.26 | 1.82 | 1.48 | 1.97 | 4.95 |  |
| Glipr2 | 0.90 | 1.02 | 0.89 | 0.89 | 0.50 | 0.73 | 3.57 |  |
| Evi2a | 1.32 | 1.53 | 1.30 | 1.11 | 0.53 | 0.81 | 1.71 |  |
| Clec4d | 2.15 | 2.25 | 1.98 | 1.43 | 1.14 | 2.52 | 4.10 |  |
| Cxcl10 | 3.77 | 2.36 | 1.83 | 1.39 | 0.94 | 2.30 | 3.06 |  |
| Cyba | 0.97 | 0.68 | 0.78 | 0.47 | 0.41 | 1.34 | 1.56 |  |
| Lrg1 | 1.46 | 1.17 | 0.98 | 0.86 | 0.82 | 1.13 | 2.08 |  |
| Tnc | 2.59 | 2.10 | 1.73 | 1.12 | 0.79 | 2.83 | 6.52 |  |
| Lmna | 0.86 | 0.62 | 1.09 | 0.46 | 0.64 | 1.13 | 2.40 |  |
| Lgmn | 1.42 | 1.21 | 1.09 | 0.97 | 0.70 | 1.71 | 3.22 |  |
| Slc11a1 | 1.20 | 1.97 | 1.97 | 1.29 | 1.18 | 1.92 | 3.79 |  |
| Tgfbi | 1.74 | 1.02 | 0.64 | 0.39 | 0.36 | 1.42 | 1.90 |  |
| Ccl8 | 2.65 | 1.44 | 1.83 | 1.08 | 1.44 | 4.47 | 4.20 |  |
| Rab11fip5 | 0.53 | 0.52 | 0.40 | 0.40 | 0.29 | 1.07 | 1.62 |  |
| Capzb | 0.82 | 0.89 | 0.75 | 0.75 | 0.90 | -2.31 | 2.52 |  |
| Cd22 | 1.11 | 1.11 | 0.86 | 0.84 | 0.97 | -0.78 | -0.90 |  |
| Srprb | -0.96 | -0.75 | 0.87 | 0.72 | -0.64 | -1.11 | 1.69 |  |
| Tbc1d22a | 0.44 | 0.46 | 0.45 | 0.30 | 0.31 | -1.50 | 0.75 |  |
| Kif1b | -0.39 | -0.43 | -0.45 | -0.41 | -0.58 | -0.93 | 2.05 |  |
| Traf6 | 1.05 | 0.71 | 1.02 | 0.62 | -0.44 | 1.45 | 2.57 |  |
| Zfp60 | -0.57 | -0.67 | -0.61 | -0.53 | -0.52 | -0.76 | 1.39 |  |
| Ankrd40 | 0.43 | 0.81 | 0.95 | 0.87 | 0.78 | -1.05 | 1.02 |  |
| Slc9a3r2 | 1.16 | 1.81 | 1.57 | 2.12 | 1.40 | -1.04 | 1.94 |  |
| Pou2af1 | 0.74 | 1.21 | 1.34 | 1.32 | 1.08 | -1.87 | -1.46 |  |
| Cd3d | 0.84 | 0.54 | 0.94 | 0.67 | 0.68 | -2.09 | -1.49 |  |
| Aif1 | 1.36 | 0.59 | 0.73 | 0.67 | 0.58 | 2.45 | 1.50 |  |
| Cd79b | 1.08 | 1.51 | 1.28 | 1.07 | 1.28 | -1.40 | -2.04 |  |
| Irf9 | 0.67 | 0.46 | 0.79 | 0.91 | 0.74 | -0.96 | 1.22 |  |
| Gpcpd1 | 0.43 | 1.06 | 1.06 | 1.14 | 0.83 | -0.74 | 2.43 |  |
| Mat2a | -0.62 | -0.38 | -0.88 | -0.33 | -0.59 | -0.91 | 1.17 |  |
| Gfpt2 | -0.49 | -0.39 | -0.61 | -0.47 | -0.58 | 1.42 | 2.73 |  |
| Rasgrp1 | 1.21 | 0.88 | 0.64 | 0.67 | 0.47 | -0.91 | -1.15 |  |
| Tpm3 | 1.03 | 0.90 | 0.72 | 0.64 | 0.49 | -1.70 | 2.48 |  |
| Cd8b1 | 0.93 | 0.71 | 0.78 | 0.73 | 0.72 | -1.93 | -1.95 |  |
| Atp2a2 | -1.16 | -1.13 | -1.02 | -0.63 | -0.65 | -1.30 | 2.32 |  |
| Acta2 | -0.76 | -0.82 | -0.79 | -0.73 | -0.50 | 0.69 | 2.49 |  |
| Elp5 | 0.48 | 0.40 | 0.47 | 0.41 | 0.49 | -1.73 | 2.64 |  |
| Slc35e1 | 0.79 | 0.90 | 0.81 | 0.92 | 0.47 | -0.72 | 2.15 |  |

**Supplemental Table 3** Characteristics of the genes included in datasets

| **Dataset ID** | **Group of samples** | **Number of detected genes** | **Number of differentially expressed genes (up/down)** | **Ranking of C1q (among the differentially regulated genes%)** |
| --- | --- | --- | --- | --- |
| **GSE37635** | **1w vs 0w** | 9781 | 3492 (1742/1750) | C1QA 48 (1.37%)  C1QB 2 (0.05%)  C1QC 19 (0.54%) |
|  | **2w vs 0w** | 9781 | 3290 (1691/1599) | C1QA 493 (14.98%)  C1QB 15 (0.46%)  C1QC 127 (3.86%) |
|  | **3w vs 0w** | 9781 | 2917 (1541/1376) | C1QA 218 (7.47%)  C1QB 19 (0.65%)  C1QC 32 (1.10%) |
|  | **4w vs 0w** | 9781 | 2443 (1368/1075) | C1QA 387 (15.84%)  C1QB 8 (0.33%)  C1QC 55 (2.25%) |
|  | **5w vs 0w** | 9781 | 1617 (877/740) | C1QA 1617 (100%)  C1QB 67 (4.14%)  C1QC 145 (8.96%) |
| **GSE97546** | IPF vs CTRL | 20141 | 10295 (9444/851) | C1QA 858 (8.33%)  C1QB 1543 (14.99%)  C1QC 242 (2.35%) |
| **GSE485** | IPF vs CTRL | 9176 | 941 (415/526) | C1QA 130 (13.81%)  C1QB 107 (11.37%)  C1QC 220 (25.71%) |
| **GSE31013** | NSCLC vs CTRL | 22073 | 4856 (1580/3276) | C1QA 1929 (39.72%)  C1QB 1410 (29.03%)  C1QC 2655 (54.67%) |

**Supplemental Table 4** Characteristics of human subjects

|  | **Control** | **IPF** |
| --- | --- | --- |
| **Number** | 6 | 10 |
| **Sex** | Male | Male |
| **Age (year)** | 57.8 ± 10.7 | 57.0 ± 5.1 |
| **Weight (kg)** | 83.2 ± 13.6 | 72.9 ± 10.3 |
| **Height (cm)** | 175.8 ± 5.9 | 172.6 ± 6.7 |
| **BMI** | 26.8 ± 3.5 | 24.4 ± 2.2 |
| **Smoking History** |  | |
| **non smoker (ns)** | 4 | 0 |
| **former smoker (fs)** | 1 | 10 |
| **current smoker (cs)** | 1 | 0 |
| **Pack year** | Fs=24 cs=39 | 22.8 ± 11.8 |
| **FEV_1_ (L)** | - | 1.99 ± 0.51 |
| **FVC (L)** | - | 2.40 ± 0.77 |
| **FEV_1_/FVC** | - | 0.85 ± 0.08 |
| **TLC (L)** | - | 3.6 ± 1.2 |
| **FEV_1_pp (%)** | - | 60.7 ± 15.5 |
| **FVCpp (%)** | - | 58.7 ± 19.7 |
| **TLCpp (%)** | - | 54.9 ± 16.9 |
| **DLCDpp (%)** | - | 27.6 ± 7.8 |

**Supplemental Table 5** The means and standard deviations of FPKM of C1q

| **Mean±SEM** | **Ctrl** | **IPF1** | **IPF2** |
| --- | --- | --- | --- |
| **C1qa** | 318.9±21.14 | 510.4±42.76 | 597.4±57.08 |
| **C1qb** | 407.8±41.26 | 558.5±59.43 | 761.9±91.36 |
| **C1qc** | 167.3±11.60 | 243.5±20.58 | 293.7±26.40 |
